# Magnetotactic advantage in stable sediment by long-term observations of magnetotactic bacteria in Earth’s field, zero field and alternating field

**DOI:** 10.1371/journal.pone.0263593

**Published:** 2022-02-24

**Authors:** Xuegang Mao, Ramon Egli, Xiuming Liu, Lijuan Zhao

**Affiliations:** 1 College of Geographical Sciences, Fujian Normal University, Fuzhou, China; 2 Institute of Geography, Fujian Normal University, Fuzhou, China; 3 Central institute for Meteorology and Geodynamics, Vienna, Austria; 4 Department of Earth and Environmental Sciences, Macquarie University, Sydney, New South Wales, Australia; Kaohsiung Medical University, TAIWAN

## Abstract

Magnetotactic bacteria (MTB) rely on magnetotaxis to effectively reach their preferred living habitats, whereas experimental investigation of magnetotactic advantage in stable sediment is currently lacking. We studied two wild type MTB (cocci and rod-shaped *M*. *bavaricum*) in sedimentary environment under exposure to geomagnetic field in the laboratory, zero field and an alternating field whose polarity was switched every 24 hours. The mean concentration of *M*. *bavaricum* dropped by ~50% during 6 months in zero field, with no clear temporal trend suggesting an extinction. Cell numbers recovered to initial values within ~1.5 months after the Earth’s field was reset. Cocci displayed a larger temporal variability with no evident population changes in zero field. The alternating field experiment produced a moderate decrease of *M*. *bavaricum* concentrations and nearby extinction of cocci, confirming the active role of magnetotaxis in sediment and might point to a different magnetotactic mechanism for *M*. *bavaricum* which possibly benefited them to survive field reversals in geological periods. Our findings provide a first quantification of magnetotaxis advantage in sedimentary environment.

## Introduction

Magnetotactic bacteria (MTB) synthesize intercellular membrane-enveloped nano-sized magnetic crystals (magnetite and/or gregeite) called magnetosomes, which are generally organized in one or more chains like a living compass [1]. In presence of an external magnetic field, MTB in water environment align and swim along the magnetic field lines [2]. This behavior, known as magnetotaxis, is expected to be advantageous for searching optimal living habitat in chemically stratified sediment, which can be realized in two ways: by reducing searching path from three dimension to one dimension [3] and/or by improving MTB sensing ability [4]. The study of magnetotactic bacteria orientation in sediment revealed that the alignment degree along the magnetic field is as low as 1% which is however sufficient for successful magnetotaxis in sediment [2]. Fossil magnetosomes (magnetofossil) have been widely found in old geological sediments [5–7]. In this case, MTB must develop capabilities or evolve to survive reversed field and weak field [8]. However, our current knowledge of how MTB endure abnormal magnetic field in natural sediment is very limited. A comprehensive investigation of magnetotaxis advantages in various magnetic field settings is the first step to understand MTB evolution in geological history.

The advantage of magnetotaxis is generally combined by specific chemotaxis (e.g. aerotaxis) to control their behaviors and distributions in a chemically stratified environment. With respect to the magneto-aerotaxis [9, 10], a north-seeking (NS) MTB cell appears defaulted NS state in oxic condition, whereas reverses its swimming direction in anoxic condition, a south-seeking state (SS). MTB cells migrate upward and downward by responding to the oxygen concentration and finally gather at the oxic-anoxic interface (OAI) where optimal growth can take place. Although magneto-aerotaxis is widely used to interpret MTB distribution and behaviors [11, 12], it is however not directly verified by wild-type MTB in the sedimentary environment [13]. Therefore, the use of magnetotaxis for maintaining MTB near the oxic-anoxic interface needs to be tested directly in sediment, because the complex combination of chemical and tactile conditions cannot be reproduced under the microscope. The simplest test that would serve this purpose is the comparison of MTB abundance in the same microcosm under different magnetic field configurations. Cancelling the Earth’s fields is served to eliminate magnetotaxis, because MTB no longer have a preferred swimming direction and they can solely rely on chemotaxis for displacement. A decrease in MTB abundance would therefore indicate that magnetotaxis effectively provided a biological advantage.

There are two types of magnetotaxis: axial magnetotaxis and polar magnetotaxis, in which the magnetic field plays a different role [14]. For axial magnetotaxis, the Earth’s field just provides a reference axis for directed swimming in both directions, as observed with the spirillum *M*. *magnetotacticum* [15]. For polar magnetotaxis, MTB cells swim only in one direction, parallel or antiparallel to the magnetic field, as observed with MC-1 cultured cocci [10]. Although diverse magneto-aerotaxis has been observed in culture MTB [10, 14], how the two types of magnetotaxis respond to a magnetic field in sedimentary environment is not yet tested. For this purpose, the polarity of a vertical or inclined magnetic field is switched at a certain rate (e.g. once a day) so that a reference axis is provided, while the field direction (in particular its vertical component) is zero on average. Such field would still support axial magnetotaxis, but not polar magnetotaxis, since during half of the cycles, when the field points upwards, the polarity of all cells make them swim away from the oxic-anoxic interface [10].

In the present study, the advantage of magnetotaxis in stable sediment was investigated by long-term observations of MTB in given magnetic field conditions: the Earth’s field, zero field and alternating field. MTB abundance and their vertical distributions in given magnetic field setups were monitored.

## Materials and methods

### Sediment preparation

**Fig 1:**
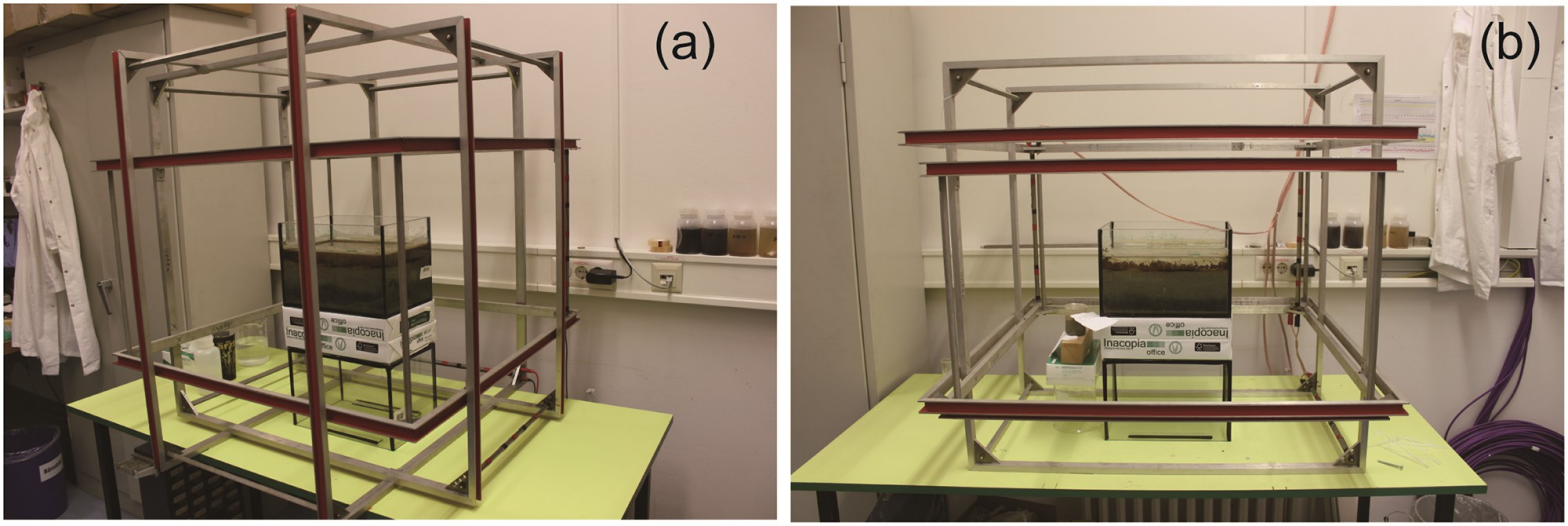
Magnetic field setups in zero field (a) and vertically alternating field (b). The zero field condition was generated by three pairs of Helmholtz coils, in which sediment microcosm was placed in the center for guarantee of near-to-zero magnetic field during the experiment. In (b), two pairs of Helmholtz coils were used to compensate external horizontal and vertical field components and the third pair was responsible for reversing vertical field component. The switch upward and downward field every 24 hours was executed by an electronically controlled commutator. The downward field intensity was 118–121.5 μT and upward field intensity was -123- -120 μT.

Top sediment from Lake Chiemsee (47°52’23” N, 12°24’25” E) of southern Germany has been collected with permission of the local authorities (Prien am Chiemsee municipality) using a bottom grab sampler and transferred into a 30×20×20 cm glass aquarium in the laboratory. Sediment was thoroughly stirred and allowed to stabilize for few months in order to reach stationary stratification (sediment microcosm in Fig 1). About 3–5 cm water was kept above the sediment: evaporation was compensated by adding distilled water. A stable oxygen gradient formed after ~5 days and was maintained unaltered for the entire experiment duration.

### Magnetic field settings

The stabilized aquarium was put at the center of three ~1×1 m Helmholtz coil pairs used to control the magnetic field over the volume occupied by the sediment microcosm (Fig 1a). The coils were connected with precision power supplies for passive field regulation. The Earth’s field in the laboratory had an intensity of ~44 μT with 71° downward inclination. Near-to-zero field conditions have been obtained by regulating the power supplies over several days in order to obtain averages close to zero. Maximum field deviations from zero mean are of the order of 1.5 μT, and tend to average out over time, so that a systematic vertical field component is not expect over a long experimental duration (S1 Text). Assuming a maximum systematic error of 0.3 μT on the vertical component, the resulting alignment of bacteria in sediment would be <1% of the typical alignment under normal conditions, making the “residual” magnetotaxis at least 100 times less effective than usual. Vertical alternating fields have been obtained with the same Helmholtz coil system and an electronically controlled commutator (Fig 1b) that switches the contacts every 24 hours, so that the field points upwards or downwards every second day.

### MTB characterization

**Fig 2:**
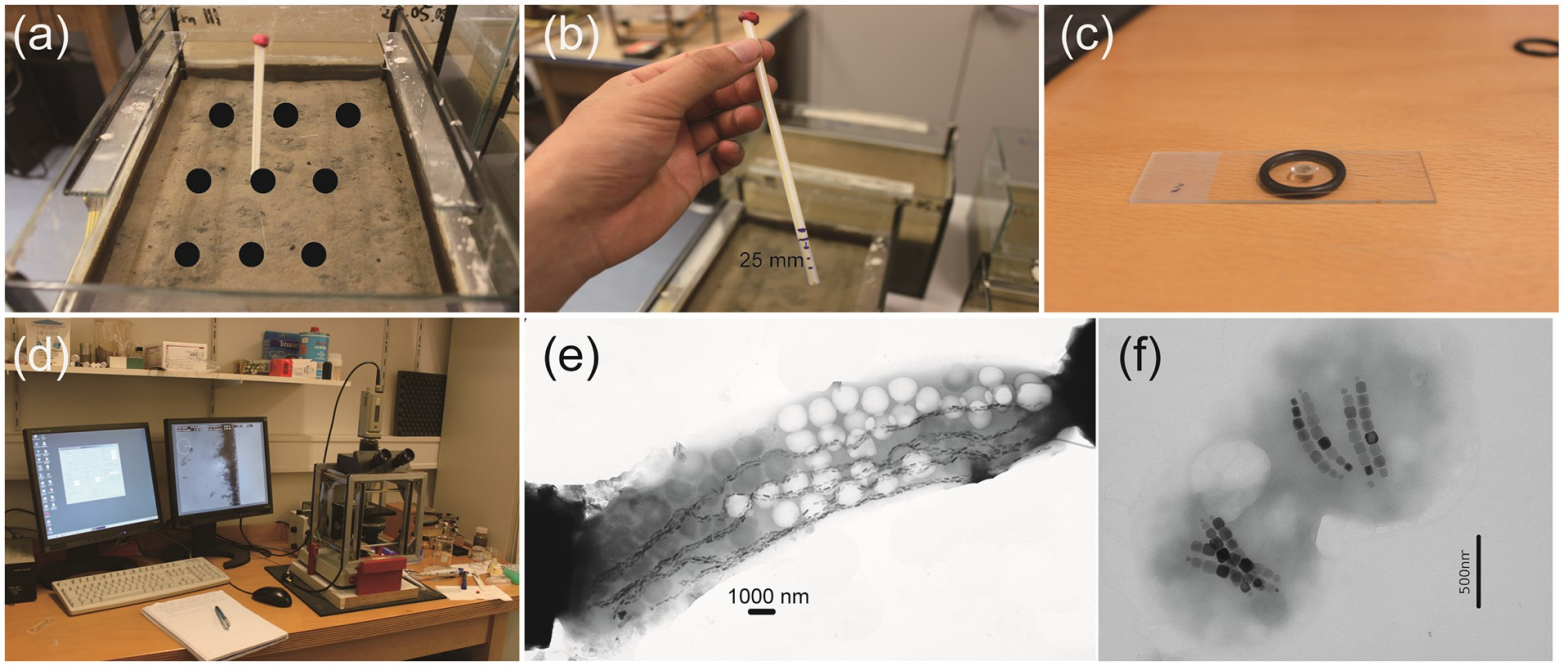
Procedures of sampling and measurements. (a) 6–9 sediment mini-cores were sampled around the 9 areas indicated by the black dots. (b) a mini-core, 25 mm long, was taken by a drinking plastic straw. Each mini-core was sliced every 1 mm increment and diluted with distilled water. (c) A drop of 10 μl diluted solution was made to be a hanging drop over an O-ring. (d) MTB number was directly obtained under the magnetodrome. (e) Transmission electron micrograph (TEM) image of a rod-shaped *M*. *bavaricum* cell with a few bundles of magnetosomes. (f) TEM image of two coccic cells with four bundles of magnetosomes in each cell.

Estimates of MTB populations and vertical distributions of cell concentration as a function of space and time in the top 2.5 cm sediment have been obtained as follows. Before and during the experiments, 6–9 homogeneously distributed sediment profiles (Fig 2a) were sampled on a regular basis (every 15 or 30 days). Sediment profiles were taken in form of mini-cores (∅ 5 mm) with a drinking straw, which easily penetrate in the topmost unconsolidated sediment (Fig 2a). After sealing the top end with plasticine, the straw was retrieved from sediment and a mini-core with ~25–30 mm length (Fig 2b) was obtained. The mini-core contained in the straw was pushed forward by applying some pressure on the sealed end of the straw, and sliced in 1 mm increments. Each slice was diluted with distilled water (200 μl) and homogenized in microtubes. 10 μl sediment solution (containing ~0.614 μl sediment) was placed on a cover slid and turned it upside down to make a hanging drop on a rubber O-ring (Fig 2c). The sample was put in magnetodrome [16, 17], an instrument consisting of optical microscope and 2 pairs of Helmholtz coils for generating a homogeneous field (Fig 2d), to let MTB move to the water/air edge in the droplet. Cells swimming out of the sediment can no longer be observed after ~20 min in a horizontal magnetic field, so that MTB were always counted after 20 min exposure to a horizontal magnetic field in the magnetodrome. Observation under the microscope confirmed the presence of abundant populations of MTB, including the rod-shaped Candidatus Magnetobacterium Bavaricum (*M*. *Bavaricum*) [2, 18, 19] (Fig 2e) and cocci (Fig 2f) with an averaged magnetic moment of 11.7×10^-15^Am^2^ and 0.01×10^-15^Am^2^ [2], respectively. These two types of bacteria provided the most stable and easy-to-observe populations: therefore, our experiments focus on the characterization of these two types of MTB.

Vertical MTB distributions were determined by averaging cell counts from 6–9 individual mini-cores as described above. Standard errors were identified with the standard deviation of the mini-cores counts at each depth. These errors are always larger than statistical counting fluctuations derived from a Poisson distribution (i.e. $\sqrt{n}$ is the error associated to *n* counts), reflecting true heterogeneities in the aquarium (S1 Text). The origin of this heterogeneities and their correlation with bioturbation is unclear. For each field setup (i.e. Earth’s field, zero field, alternating field), average profiles have been calculated from all sampling dates in order to determine the equilibrium population established under these conditions. Because of the large temporal fluctuations sometimes observed in twin aquaria kept under identical conditions, we verified mean MTB populations in a twin aquarium constantly exposed to the Earth’s field at the time when the ~6 month zero-field experiment was ending.

The method described above for quantifying MTB populations in sediments might underestimate the real number of cells in the sediment, because there is no mean to verify that all cells are active and swim out of the sediment in the hanging drop assay. However, the cell counting procedure was identical for all experiments, so that any variation is attributable to a true change in the population of active MTB in sediment.

### MTB population evolution as the reformation of chemical stratification

**Fig 3:**
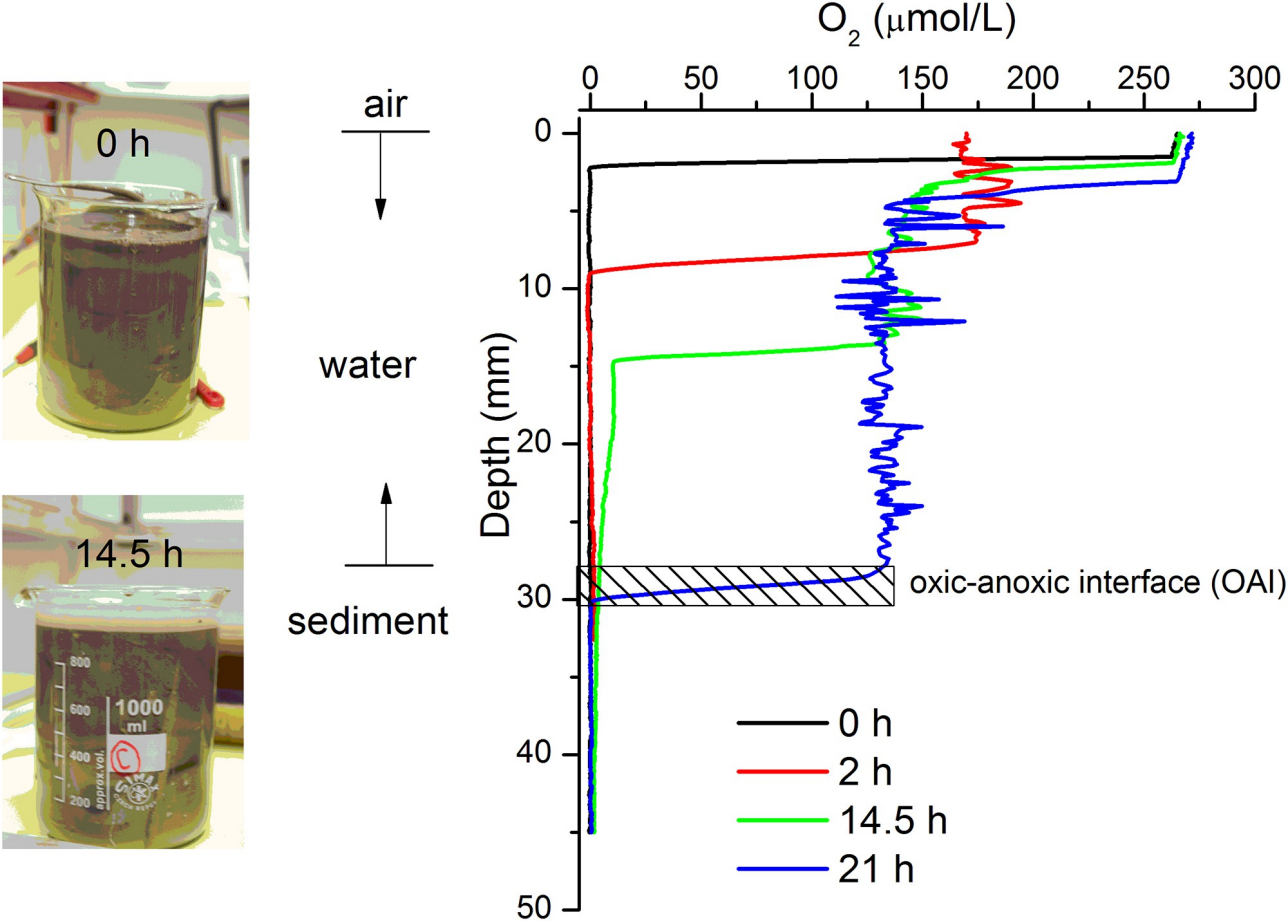
Reformation of oxygen gradient in a disturbed sediment. The sediment in a glass baker was stirred completely to form sediment slurry (1000 ml, left top). Let the sediment stabilize until a clear water-sediment interface was reached (e.g. at 14.5 hours) meanwhile oxygen profile was measured continuously from the beginning (0 hour) to reformation of stable oxygen gradient (21 hours). The contour indicated the oxic-anoxic interface which was generally stabilized in the following experiments.

In order to monitor the formation of stable MTB populations after the preparation of a sediment-filled aquarium, we used the same sediment material to prepare a microcosm. Preparation started with filling a 1000 ml glass baker with homogenized sediment slurry (Fig 3). Oxygen profiles have been measured in water and sediment with a microprofiling system from UNISENSE (www.unisense.com), consisting of a computer-controlled vertical stage equipped with an oxygen microsensor (OX50) with 50 μm external tip diameter and 0.3 μM detection limit. Initial O_2_ measurements revealed anoxic conditions in the entire suspension, due to rapid oxygen consumption by sediment previously coming from an oxygen-free environment. A steep oxygen gradient forms and moves down in the water column within the first few hours (Fig 3), until it reaches the sediment-water interface after ~14.5 hours. The initial fast progress of the OAI in the water column (~1.2 mm/h) comes to a halt as soon as the sediment-water interface is reached. MTB profiles have been measured at regular intervals, first on a daily basis (day 2 until day 6), then every two days until day 10, every four days until day 18, and then occasionally until day 123.

## Results

### Initial evolution of MTB populations in the Earth’s field

**Fig 4:**
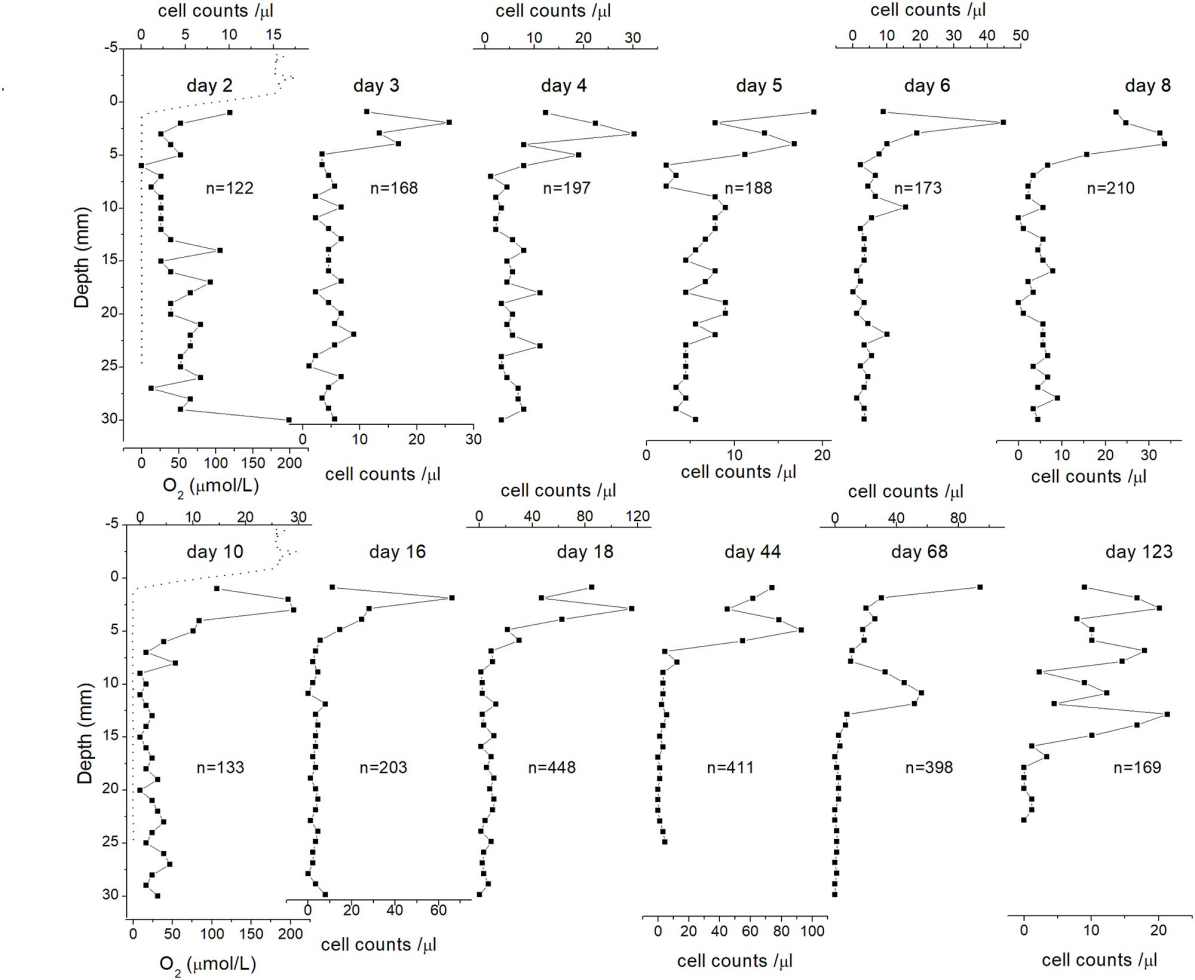
Vertical distribution changes with time. Development of *M*. *bavaricum* vertical distribution (square) and oxygen gradient (dotted line).

**Fig 5:**
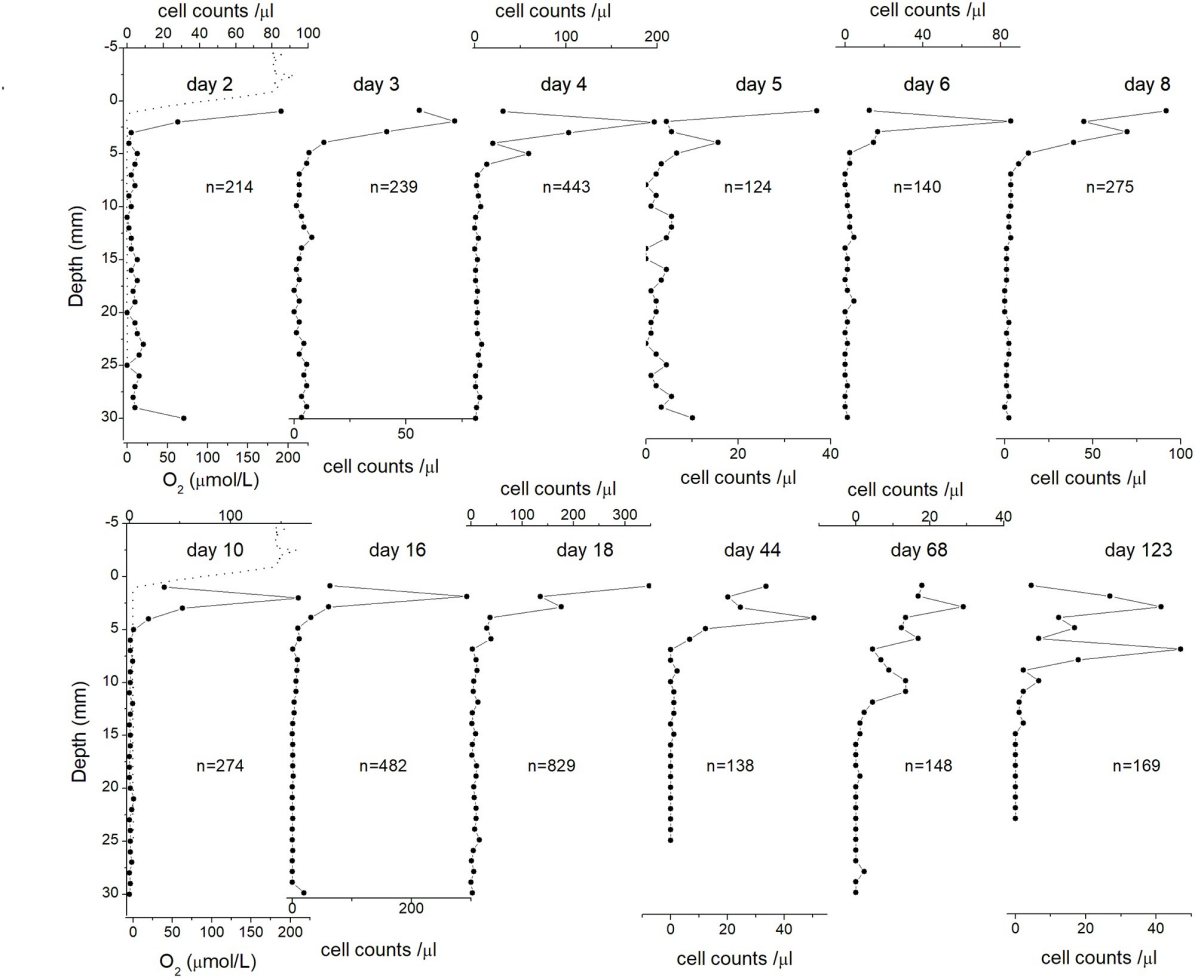
Vertical distribution changes with time. Development of cocci vertical distribution (square) and oxygen gradient (dotted line).

Figs 4 and 5 show the vertical distribution of *M*. *bavaricum* and cocci, respectively with time. During the first 2 days of the newly formed sediment column of the microcosm (day 0 to day 2), MTB were scattered at all depths, with an incipient formation of a peak in the topmost 2 mm. This peak, located exactly at the OAI, developed steadily during the next day, producing a population increase of both *M*. *bavaricum* and cocci. As far as cocci are concerned, the peak increased in amplitude until day 4, and was followed by a sudden population drop with no significant change in depth distribution. A second increase, always in form of a sharp peak at the OAI, was observed at day 18. Interestingly, the population of cocci tended to spread out into larger depths from day 44 on, although no changes of the oxygen gradient have been observed since day 16. A similar evolution occurred for *M*. *bavaricum*, which reached its first population maximum at day 18, with an unusually shallow depth distribution within 1–2 mm from the OAI that has never been observed again at a later point. After this first population maximum, a second peak developed at ~13 mm depth on day 68, and the depth distribution remained wider at later points.

Overall, even taking into account unavoidable heterogeneities between profiles taken at different points in the microcosm, the initial evolution of MTB populations can be outlined. Initially, cells are scattered at all depths, reflecting the homogeneity of the initial slurry. 2–3 days after the OAI has penetrated the sediment, a first sharp MTB peak develops within 2 mm from the OAI. An initial increase of MTB population occurs entirely in this peak for both *M*. *bavaricum* and cocci, while the homogeneous cell concentration at depths remains constant. The OAI-related peak eventually reaches a first maximum at day 4 for cocci and day 18 for *M*. *bavaricum*. A second maximum is reached by cocci at day 18, always within 2 mm from the OAI. Until this point, the depth distribution of both MTB types conforms to the classical picture of population maxima at or just below the OAI. From day 18 on, however, MTB populations start to spread over greater depths with no change of the OAI depth, and sometimes a second peak clearly appears ~10 mm below the OAI. The reason for this change is not clear; however, MTB profiles taken after ~1 month from microcosm formation do not show any systematic changes in time. Random fluctuations in depth distribution are expressed by the transient development of peaks located either 1–3 mm from the OAI, or ~6–10 mm deeper.

### Total MTB population in the Earth’s field, zero field and alternating field

**Fig 6:**
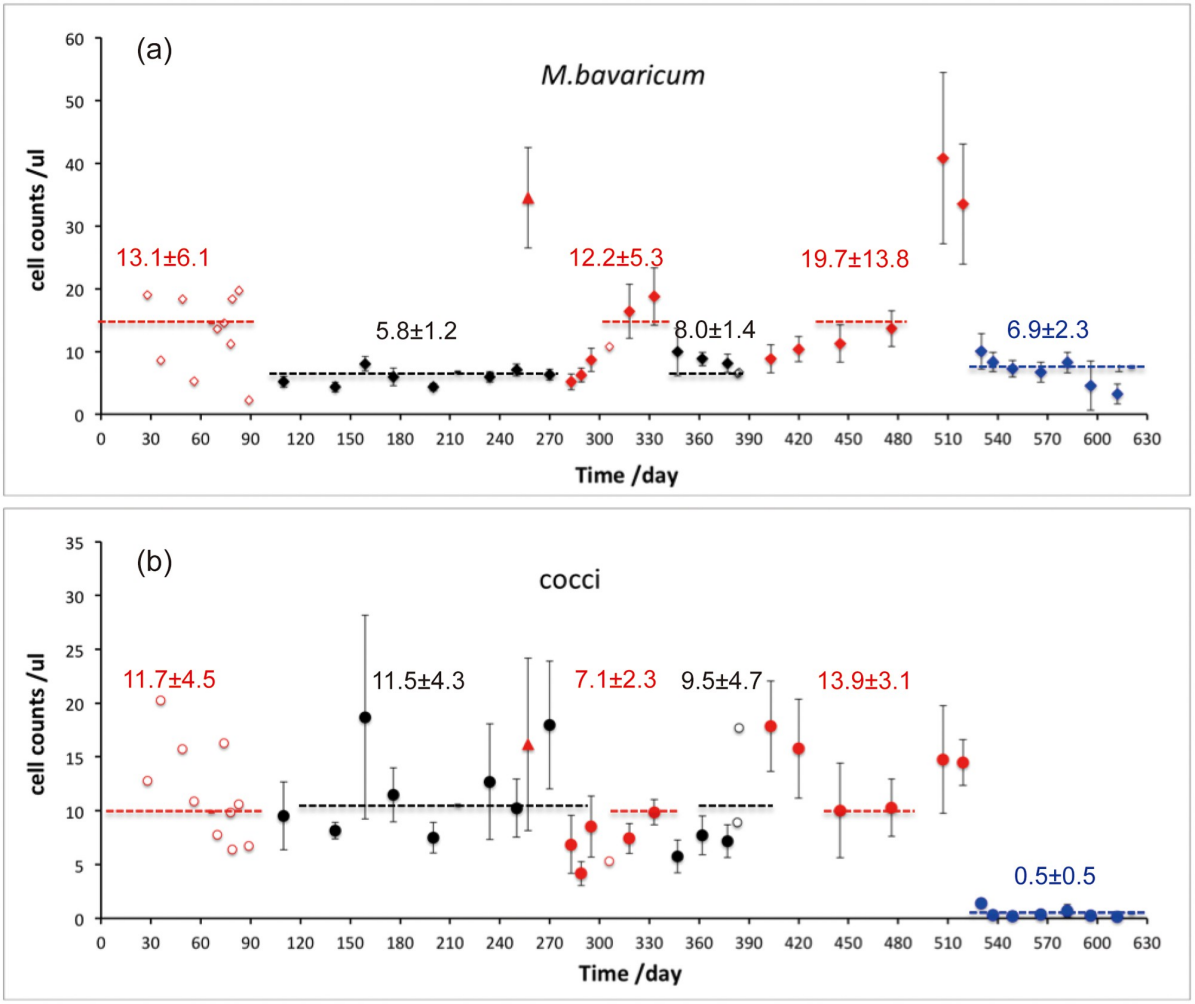
Population changes of *M*. *bavaricum* (a) and cocci (b) in Earth’s field (red symbols), zero field (black symbols) and alternating field (blue symbols) as a function of time. Filled symbols represented averaged population density of 6–9 profiles (25 mm long) and smaller open symbols represented population density of single profile. Horizontal dashed lines and numbers errors referred to mean concentration in zero field (black), Earth’s field (red) and alternating field (blue). Red filled triangle represents one sediment microcosm constantly in Earth’s field.

MTB population changes in Earth’s field, zero field and alternating field are shown in Fig 6a. *M*. *bavaricum* decrease from 13.1 ± 6.1 cells/μl in Earth’s field to 5.8±1.2 cells/μl during the following 6 months in zero field. The population recovered to the initial value 12.2 ±5.3 cells/μl in Earth’s field for 1.5 months and decreased 8.0± 1.4 cells/μl in zero field. The similar decrease of population density also occurred to alternating field in which *M*. *bavaricum* decreased to 6.9±2.3 cells/μl. Interestingly, *M*. *bavaricum* in zero field and alternating field did not indicate extinction even over 6 months in the zero field and 3 months in the alternating field.

Cocci (Fig 6b) displayed large variation with population density 11.2±4.4 cells/μl in Earth’s field and 10.7±4.4 cells/μl in zero field. The population difference between Earth’s field and zero field can be tested by statistical analysis. According to two-sample Kolmogorov-Smirnov test with null hypothesis, based on two datasets: single profiles in Earth’s field and zero field, the null hypothesis (i.e. two datasets are same) is rejected at 85% confidence level (S1 Text). If using averaged population density (S1 Table) in Earth’s field and zero field, the null hypothesis (i.e. two datasets are same) is rejected at 66% confidence level. Therefore, the two datasets (i.e. in Earth’s field and zero field) can be seen same with high confidence level. Cocci in alternating field experienced a dramatic decrease from 13.9±3.1 cells/μl in the Earth’s field to 0.5±0.5 cells/μl, to almost extinction.

### Vertical distributions

**Fig 7:**
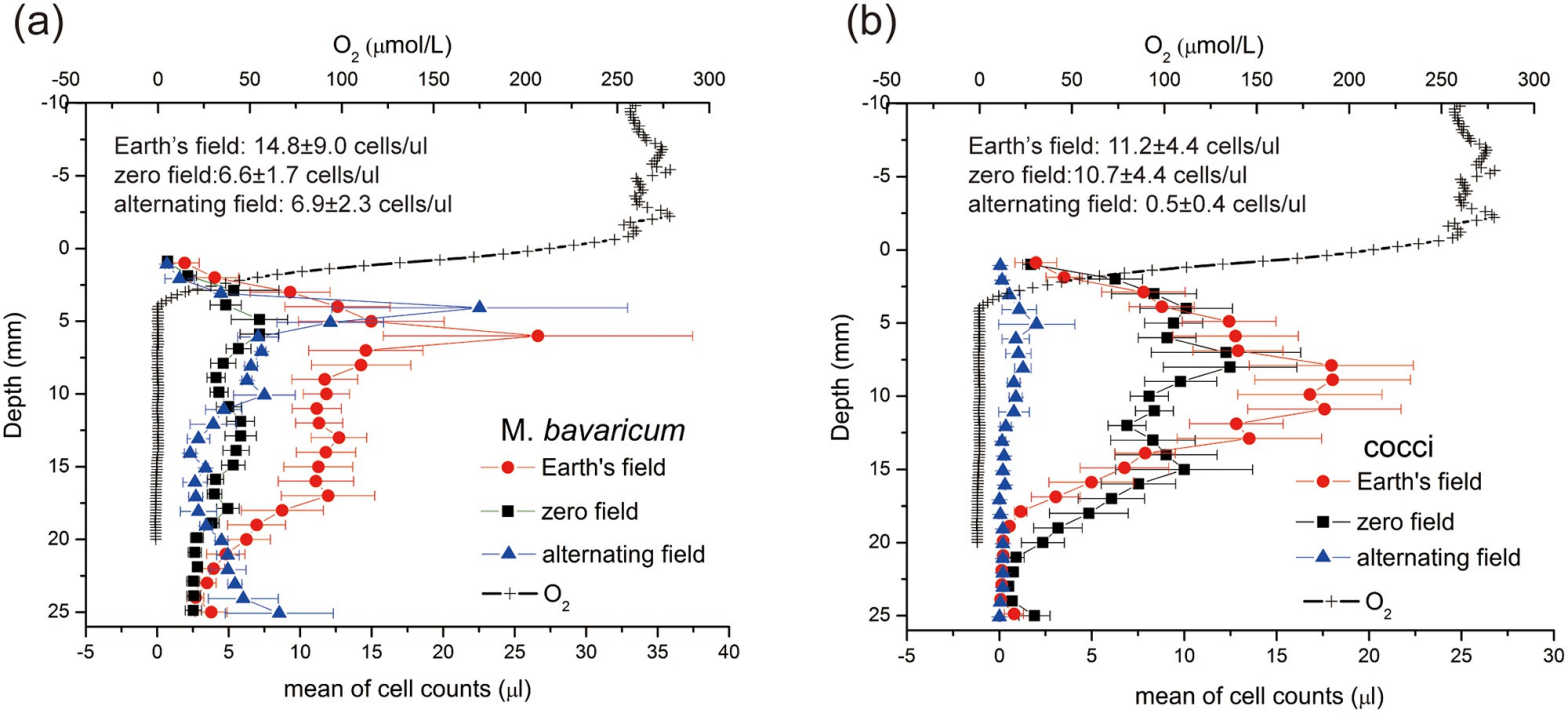
Vertical distributions in the three field settings. Vertical distributions (average of each field setting) of *M*. *bavaricum* (a) and cocci (b) in Earth’s field, zero field and alternating field. The population intensity (cells/μl) in three field settings was inserted.

Depths distributions on average of *M*. *bavaricum* and cocci corresponding to a given field condition are shown in Fig 7. In the Earth’s field, *M*. *bavaricum* occurs over a slightly larger range of depths (i.e. 1–25 mm), as also reported by Jogler et al. (2010) [20], however, its depth distribution has a bimodal character (Fig 7a red filled circles), with a first, usually more pronounced peak at ~6 mm depth, and a second, broader one, at 13–17 mm depth. The vertical distribution of cocci in the Earth’s field is a unimodal function staring within ~1 mm from the sediment-water interface (Fig 7b red circles), where oxygen concentration is ~50% of saturation, and peaking at 7–11 mm depth, 3 mm below the level where O_2_ drops below measurable levels. The maximum depth for the occurrence of cocci is ~20 mm. Despite the presence of MTB at the sediment-water interface, MTB cells could never be detected in the water column. Overall, MTB occur within, and especially below the oxic-anoxic interface (OAI) as observed in other freshwater microcosms [21, 22], marine sediment [23], as well as eutrophic water columns [24, 25].

Average profiles of cocci taken in zero-field are similar to profiles in the Earth’s field and only slightly wider in the deeper range (Fig 7b black squares). The distribution appears slightly bimodal, due to some individual profiles with strongly bimodal character; however, this feature is not a systematic characteristic of cocci in zero field. The depth distribution of *M*. *bavaricum* does also not appear to change significantly in zero field (Fig 7a black squares), except for a clear, proportional concentration decrease at all depths. Cocci profiles obtained in the alternating field are characterized by drastic decrease of cell concentration (Fig 7b blue triangles), with few cells occurring only in a limited depth range closer to the OAI (i.e. 1–13 mm). The depth distribution of *M*. *bavaricum*, on the other hand, becomes more evidently bimodal (Fig 7a blue triangles), with the upper peak moving up by 2 mm, and a lower peak forming at >24 mm depth. The cell concentration drops to almost zero at intermediate depths around ~15 mm.

## Discussion

### Magnetotactic advantage

The direct test of magnetotactic advantage is to cancel the magnetic field. A clear effect of zero field conditions can be observed with *M*. *bavaricum*, whose concentration decreased by a factor ~2 immediately after the beginning of the experiment (Fig 6a). The mean concentrations appeared to remain constant over the ~6 months experiment duration, without evidence for any further decrease. After re-establishing normal Earth’s conditions, mean concentrations increased slowly during ~1.5 months. A second experiment with zero-field conditions produced a similar concentration drop. Several explanations for these observations are possible, as summarized in the following models.

1. True population decreases. In this case, we assume that cell counts with the hanging drop assay reflect, or are at least proportional to, the real concentration of cells in sediment. The sudden concentration decreases at the onset of zero-field conditions, and the slow recovery as the Earth’s field was re-established, can be interpreted as the consequence of magnetotactic advantage removal. *M*. *bavaricum* might continue surviving indefinitely relying on chemotaxis as other bacteria do. However, the lack of preferred cell orientation produces a three-dimensional random walk pattern with substantially longer times and higher energies required to obtain macroscopic displacements in sediment.
2. Cells become non-motile. In this case, we assume that the true cell concentration of *M*. *bavaricum* in sediment did never change systematically, and that the apparent 50% drop observed with the hanging drops assay during zero-field conditions is due to the fact that 50% of the cells become non-motile. Slow apparent population recovery at the end of the experiment is due to an increasing number of cells becoming motile again. The reason for motility loss could be related to the lack of correlation between cell magnetotactic polarity (i.e. swimming direction), and change of environmental conditions. This concept can be clarified with the following example. Consider a cell with polar magnetotaxis under normal field conditions, which is located above the OAI, therefore exposed to high oxygen (HO) concentrations. In this case, according to the model of Frankel et al., (1997), the cell will be north-seeking (NS), swimming downward to greater depths until low oxygen (LO) concentrations are reached. If magnetic field is removed, the cell will still be NS, but will swim at random, continuing to remain exposed to HO conditions for much longer time. If unfavorable (in this case HO) conditions will persist beyond a certain amount of time–within which better conditions are usually found with magnetotaxis–the cell might enter into an ‘emergency state’ and become inactive, until casual events such as bioturbation will eventually bring it again into a more suited environment. Lack of a magnetic field will therefore increase the number of cells that enter into this state at any time. This hypothesis has an important drawback, because it assumes that the MTB population remains constant while 50% of individual cells are in such unfavorable conditions that they lose motility–but do not die. This endurance capability might be attributed to *M*. *bavaricum* on account of its unusual size and thick external membrane [26], the situation by which 50% of the cells loose motility appears as clear disadvantage.
3. Degeneracy. This scenario is based again on the assumption that the true MTB concentration in sediment remained constant, but some cells degenerate and no longer synthesize magnetosomes. This kind of degeneracy is observed in old MTB cultures, where fewer and fewer cells have a measurable magnetic moment [27]. In this case, unlike cultures, degeneracy is induced by the lack of a magnetic field: if a random mutation produces non-magnetic cells with intact chemotactic capabilities, these cells have exactly the same survival probability as their magnetic counterparts. An increased number of non-magnetic cells will result in an apparent decrease of MTB concentration in sediment, because these cells are no longer counted in the hanging drop assay.

Models (2) and (3), although rejecting a population decrease in zero-field conditions, actually imply a magnetotactic advantage. In (2), loss of motility is induced by unfavorable conditions, which are a direct consequence of magnetotaxis lack. In (3), it is assumed that non-magnetic cells suffer from some sort of disadvantages under normal field conditions, so that magnetotaxis is actually an advantage. Therefore, all three models for explaining the zero-field experiment imply that there is a magnetotactic advantage for *M*. *bavaricum* in sediment.

While the existence of a magnetotactic advantage for *M*. *bavaricum* in sediment is proved by the zero-field experiment, the apparent insensitivity of cocci to the cancellation of magnetotaxis is puzzling. A possible explanation for the difference between the two types of bacteria comes from the activity of individual cells within the preferred depth range in sediment. While MTB populations as a whole were already living at preferred depths at the beginning of the zero-field experiments, therefore not needing to move from there, individual cells within the same depth distribution might require changing depth according to their internal state. In this case, the observed depth distributions could be a dynamic equilibrium of cells moving up and down between upper and lower limits. This hypothesis is particularly well suited for explaining the bimodal distribution of *M*. *bavaricum* (Fig 7a), and the independent development of two peaks in newly formed microcosms (Fig 4).

In fresh microcosms prepared from homogenized slurry, sediment taken at any depth contains particles with compositions corresponding to a wide range of depths in the original sedimentary column (Fig 3). Therefore, the same combination of nutrients can be found everywhere during initial formation of a new microcosm, and the only factor influencing the depth distribution of MTB is the oxygen gradient. Indeed, the initial depth distributions of cocci and *M*. *bavaricum* are identical and tightly concentrated at the OAI (Figs 4 and 5). This stage produces a first MTB “bloom”, and, in case of cocci, a second one, with an increase in total population by a factor 2–4. Once the “bloom” is over (day 44 in Fig 5) a second population peak develops at greater depths, ~10 mm below the first one, without any change of the oxygen concentration profile. From this point on, the depth distributions of *M*. *bavaricum* and cocci extend ~20 mm and 15 mm below the OAI, respectively. Especially in case of *M*. *bavaricum*, individual profiles taken in mature microcosm often display the shallower or the deeper population peak in a clear manner. These observations can be interpreted as population oscillations at given depths, with each peak signaling a localized “blooming”, or as a migration of cells within the usual range of living depths.

Vertical ‘shuttling’ could satisfy specific metabolic requirements related to substances that are usually not found at same depth in stratified environments (except for freshly prepared, homogenized microcosms). This hypothesis is particularly appealing for *M*. *bavaricum*, given the observation that some cells contain filled sulfur inclusions and appear darker than other, ‘empty’ cells [12]. Dissolved sulphides could not be detected in similar microcosms hosting *M*. *bavaricum* [20]; however, *M*. *bavaricum* could obtain sulfur from solid phases in deep sediment [12]. In this case, the typical cycle of individual cells could consist in a ‘deep’ phase, where sulfur is accumulated inside the cell, followed by a ‘shallow’ phase where the incorporated sulfur is oxidized. One could in this case expect a correlation between the proportion of dark cells and depth in sediment, which could never be observed. However, in case of a stationary, dynamic ‘shuttling’ between two depth ranges, both ranges would contain equal amounts of empty cells (i.e. just arriving or just leaving) and full cells (i.e. just leaving or just arriving).

If the vertical ‘shuttling’ hypothesis applies to *M*. *bavaricum*, and to a lesser extent, or not at all, to cocci, it can explain the experimental results in zero field. Lack of magnetotaxis makes vertical ‘shuttling’ rely exclusively on chemotaxis, with increased energy costs, and it becomes obvious that MTB performing such ‘shuttling’ are more affected by zero-field conditions than MTB cells that tend to maintain a constant depth, such as cocci. This hypothesis, if verified, provides interesting insights into MTB metabolism.

### Axial and polar magneto-aerotaxis

Frankel et al. (1997) observed two types of magneto-aerotaxis. Axial magneto-aerotactic MTB (e.g. the spirillum *M*. *magnetotacticum*) sense oxygen concentrations continuously while moving, thereby perceiving a temporal [O_2_] increase or decrease (temporal sensory mechanism). The swimming direction is almost instantaneously controlled by the temporal trend in oxygen concentration: if [O_2_] increases, cells are leaving the OAI by swimming upwards, and the swimming direction is soon reversed. In this case, the cell will swim downwards and sense a [O_2_] decrease, in which case it will change swimming direction again. Axial magneto-aerotaxis is thus characterized by a continuous change of swimming direction around the OAI. Field polarity is indifferent, because cells determine their swimming direction on the basis of a sensed chemical gradient. Indeed, field reversal applied to this type did not change cell stratification. On the other hand, polar magneto-aerotactic cells sense [O_2_] and determine their swimming direction with respect to the magnetic field according to a threshold mechanism. As long as [O_2_] is above an upper critical threshold (HO), cells are presumably located above the OAI and will consistently swim along a direction that brings them further down. As cells continue to swim downwards, they eventually cross the OAI and, at a later point, a lower critical [O_2_]-threshold (LO). As soon as sensed [O_2_] drops below this threshold, cells will reverse their swimming direction. This mechanism gives a consistent swimming direction with respect to the magnetic field for the HO and LO states. If the magnetic field is reversed, HO-cells, which are already above the OAI, will swim upward, and LO-cells, which are already below the OAI, swim downwards. In both cases, cells swim away from the OAI and disperse, as observed with MC-1 in water.

Polar and axial magneto-aerotaxis can be distinguished in the hanging drop assay [14]. Recalling that this assay is performed in normal atmosphere, and that the water drop is soon saturated with oxygen, polar magnetotaxis with MTB from the Northern hemisphere is manifested by consistent NS swimming, because cells are in HO state (i.e. ‘above’ the OAI). On the other hand, axial magnetotaxis, which is based on temporal sensing of oxygen gradients, does not produce a consistent swimming direction, because uniform saturation with O_2_ produces a random signal that makes cells change their swimming direction continuously and oscillate back and forth. According to the hanging drop assay, both cocci and *M*. *bavaricum* perform polar magneto-aerotaxis. When these MTB reach the northern edge of the drop, they display different swimming pattern, i.e. ‘ping-pong’ motion for cocci and short excursions performed with backward swimming. These, however, appear to be tactile responses, since freely swimming cells are consistently NS before reaching the edge of the water drop. Attempts to observe SS cells in their LO state, however, failed systematically [13], raising some questions about the validity of the polar magneto-aerotaxis model for uncultured bacteria.

Experiments in alternating field can probe the existence of polar and axial magneto-aerotaxis directly in sediment. Field direction switching will not affect axial magneto-aerotaxis, as discussed above, while it transforms magnetotaxis advantage into a disadvantage in case of polar magneto-aerotaxis. Our results with cocci exposed to an alternating field support the hypothesis that they perform polar magneto-aerotaxis, because cell number experienced a rapid drop close to a complete extinction. Interestingly, the residual cell population appears more concentrated around the OAI (Fig 7b), while the opposite would be expected by cells swimming in the wrong directions, as seen with MC-1 in water [10]. This result can be interpreted in terms of better survival chances near the OAI. Again, as discussed for the zero-field experiment, the cell concentration drop could be apparent, if cells become non-motile as consequence of prolonged exposure to LO or HO states.

*M*. *bavaricum*, on the other hand, is affected by alternating fields in a similar manner as with zero fields in terms of population decrease. Interestingly, the bimodal nature of its depth distribution becomes more pronounced, with a ~2 mm upward shift of the upper peak, and a >5 mm downward shift of the lower peak. The divergence of the two peaks can be explained with polar magneto-aerotaxis; however, the persistence of an apparently stable population indicates some fundamental differences with cocci. Two possibilities are discussed in the following:

1. After a field reversal, *M*. *bavaricum* cells move in the wrong directions, spreading the upper and lower population peaks. The situation is reversed during normal polarity periods, so that the divergence of the two peaks does not continue indefinitely. This situation, however, impedes successful cell migration over long distances. The persistence of a stable, although reduced, population might be due to the capability of *M*. *bavaricum* to endure adverse conditions for long periods of time. In this case, longer experiments in an alternating field should be performed to see if the population finally declines.
2. *M*. *bavaricum* can ‘switch’ its polar magneto-aerotaxis under critical conditions [28], as explained with the following example. Consider a cell in its LO state in sediment from the Northern hemisphere. At some point, the cell becomes SS in order to move up in sediment. Its LO state will end soon under normal conditions. On the other hand, the same action in a reversed field brings the cell further down in the sediment and its LO state persists. At a certain point, the cell might switch its polarity mechanism, becoming NS in a LO state. Switched cells would therefore be able to use magnetotaxis in the reversed field. Because the Earth’s field does not reverse during the lifetime of individual cells, the evolutionary advantage of such capability is unclear. A possible explanation is based on the presence of strong, localized chemical gradients, for example around decomposing organic matter. In this case, cells located right below such localized gradients would need to reverse their polar magneto-aerotaxis in order to successfully exploit such gradients.

No matter which possibility actually occurs to *M*. *bavaricum*, the potential capability of enduring adverse field or switching the polar magneto-aerotaxis must help them survive field reversals in geological periods. Phylogenic analysis revealed that *M*. *bavaricum* cells were much older than other MTB [20, 29] even possibly dated back to Archean [30]. MTB must develop capabilities to survive adverse field conditions, for example reversed field and weak field during field reversal. The endurance of *M*. *bavaricum* to the zero field and alternating field demonstrated in the present study, although not comparable with geological time scale, likely have improved their survival possibility throughout the geological periods. The different responses of *M*. *bavaricum* and cocci to given field settings infer that magnetotaxis might play a species-specific role, which aids to regulate the MTB community in given living habitats.

It should be noted that although the present study provides a first quantification of magnetotaxis advantage in sedimentary environment, the physical mechanism behind these phenomena is far less understood. Further work is worthful to test the hypothesis proposed in the present study, for example, a test of vertical shuttling of *M*. *bavaricum* and a longer duration of experiment in given field setups.

## Conclusions

In order to understand magnetotactic advantage in sediment, sediment microcosms in rich of two wild-type MTB (*M*. *bavaricum* and cocci) were imparted to Earth’s field, zero field and alternating field as long as 612 days. Compared to that in the Earth’s field, the population of *M*. *bavaricum* drops by 50% in the zero field with no trend of further decrease or extinction as long as 6 months, and the vertical distribution pattern hardly changed. Temporal fluctuations in cell concentrations can always be interpreted as true population fluctuations or fluctuations in the proportion of motile or magnetic cells. It indicated the existence of a magnetotactic advantage for *M*. *bavaricum*, while seemed ambiguous for cocci, because cocci population hardly changed in zero field. This difference might be attributable to vertical shuttling which applies to M. *bavaricum* but not to cocci. The evolution of MTB populations and depth distributions in freshly prepared microcosms strongly support the hypothesis that individual cells, especially in the case of *M*. *bavaricum*, might ‘shuttle’ within a certain depth range in order to satisfy different metabolic requirements.

Cocci in the alternating field nearly went extinct which conformed to our current knowledge about polar magneto-aerotaxis. *M*. *bavaricum* in alternating field dropped by 50% with no trend of extinction as that in zero field and led to divergence of the bimodal distribution. *M*. *bavaricum* appeared capable of enduring periods of reversed field polarity when magnetotaxis became disadvantageous, which might help them survive field reversal in geological periods.

## Supporting information

S1 Table This table contains all experimental data underlying Figs 3–7. (XLSX)

S1 File Field settings in zero field and data statistics. Section 1: zero field settings. Section 2: example of spatial variation. Section 3: Kolmogorov-Smirnov test accompanied with null hypothesis.
(DOCX)
