# Supplementary material for: Magnetotactic advantage in stable sediment by long-term observations of magnetotactic bacteria in Earth’s field, zero field and alternating field
Source: PLoS One. 2022 Feb 24;17(2):e0263593. doi: 10.1371/journal.pone.0263593 (PMC8870540; doi:10.1371/journal.pone.0263593)
Supplement: S1 File — Section 1: zero field settings. Section 2: example of spatial variation. Section 3: Kolmogorov-Smirnov test accompanied with null hypothesis. (DOCX) [file pone.0263593.s002.docx]

**Magnetotactic advantage in stable sediment by long-term observations of magnetotactic bacteria in Earth’s field, zero field and alternating field**

Xuegang Mao^1,2*^, Ramon Egli^3^, Xiuming Liu^1,2,4^, and Lijuan Zhao^1^

**1** College of Geographical Sciences, Fujian Normal University, Fuzhou 350007, China, **2** Institute of Geography, Fujian Normal University, Fuzhou 350007, China, **3** Central institute for Meteorology and Geodynamics, Hohe Warte 38, 1190 Vienna, Austria. **4** Department of Earth and Environmental Sciences, Macquarie University, Sydney NSW 2109, Australia.

*Corresponding author: Xuegang Mao, maoxuegang1@163.com

**Supporting information**


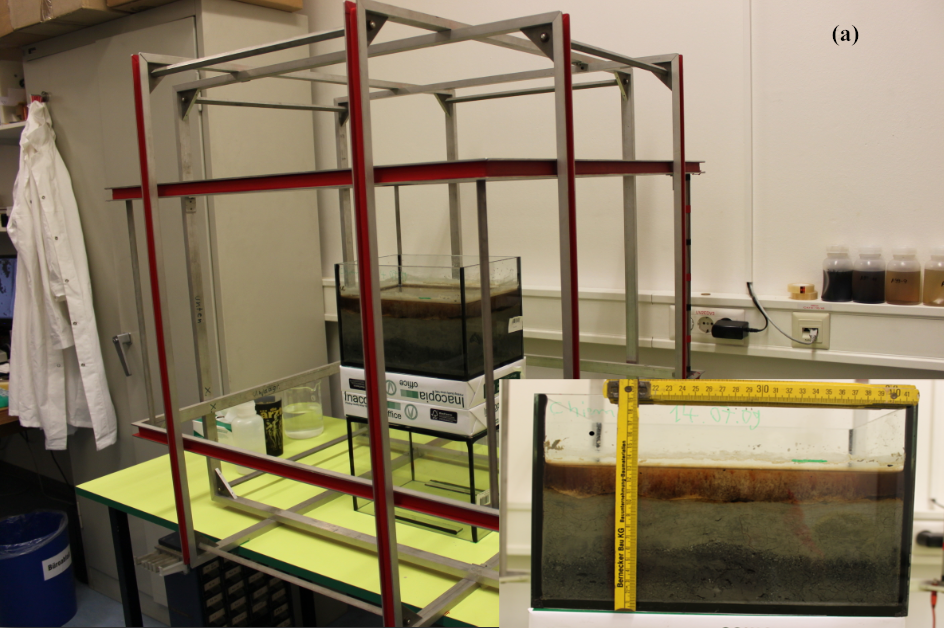


X

Y

Z


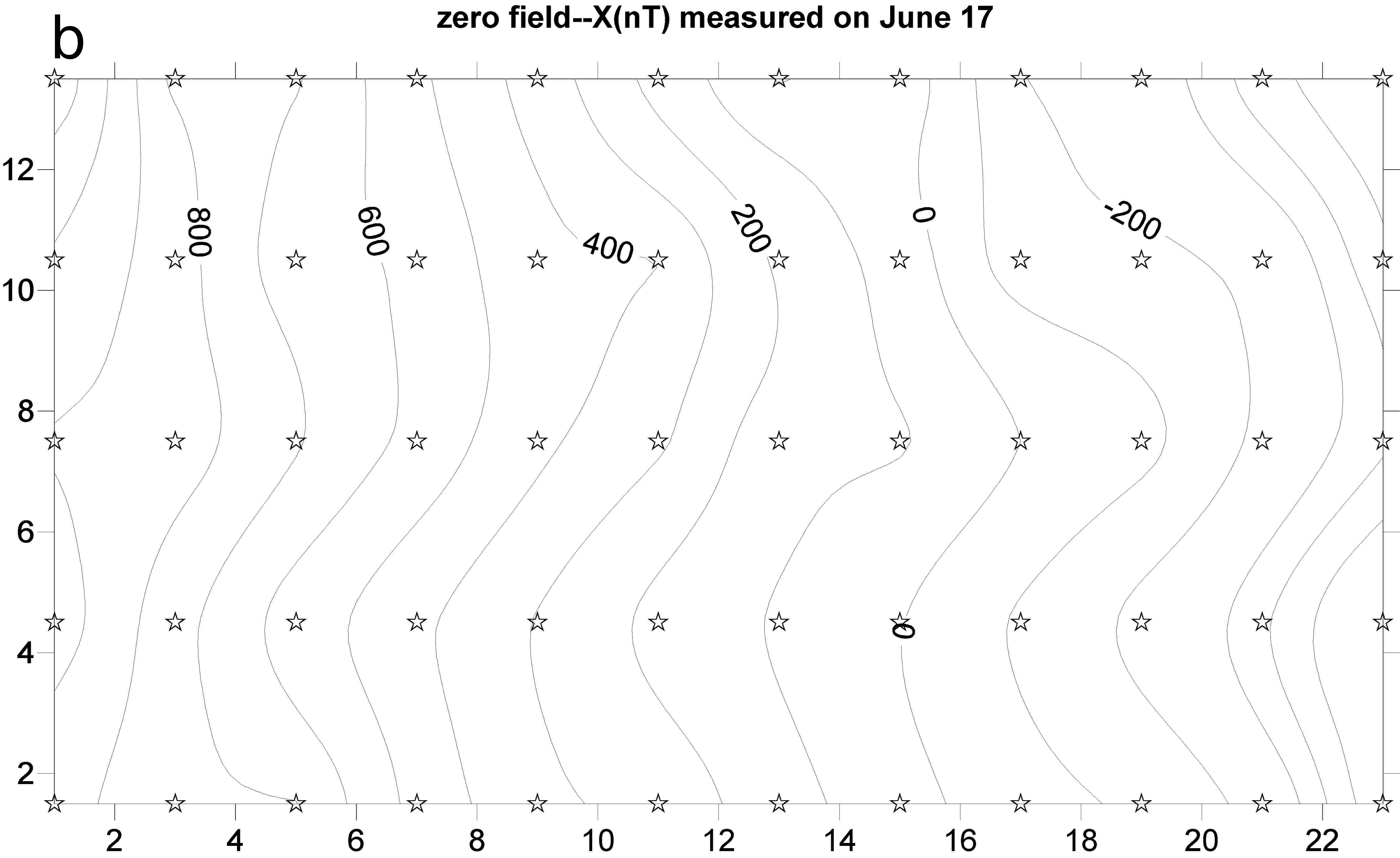

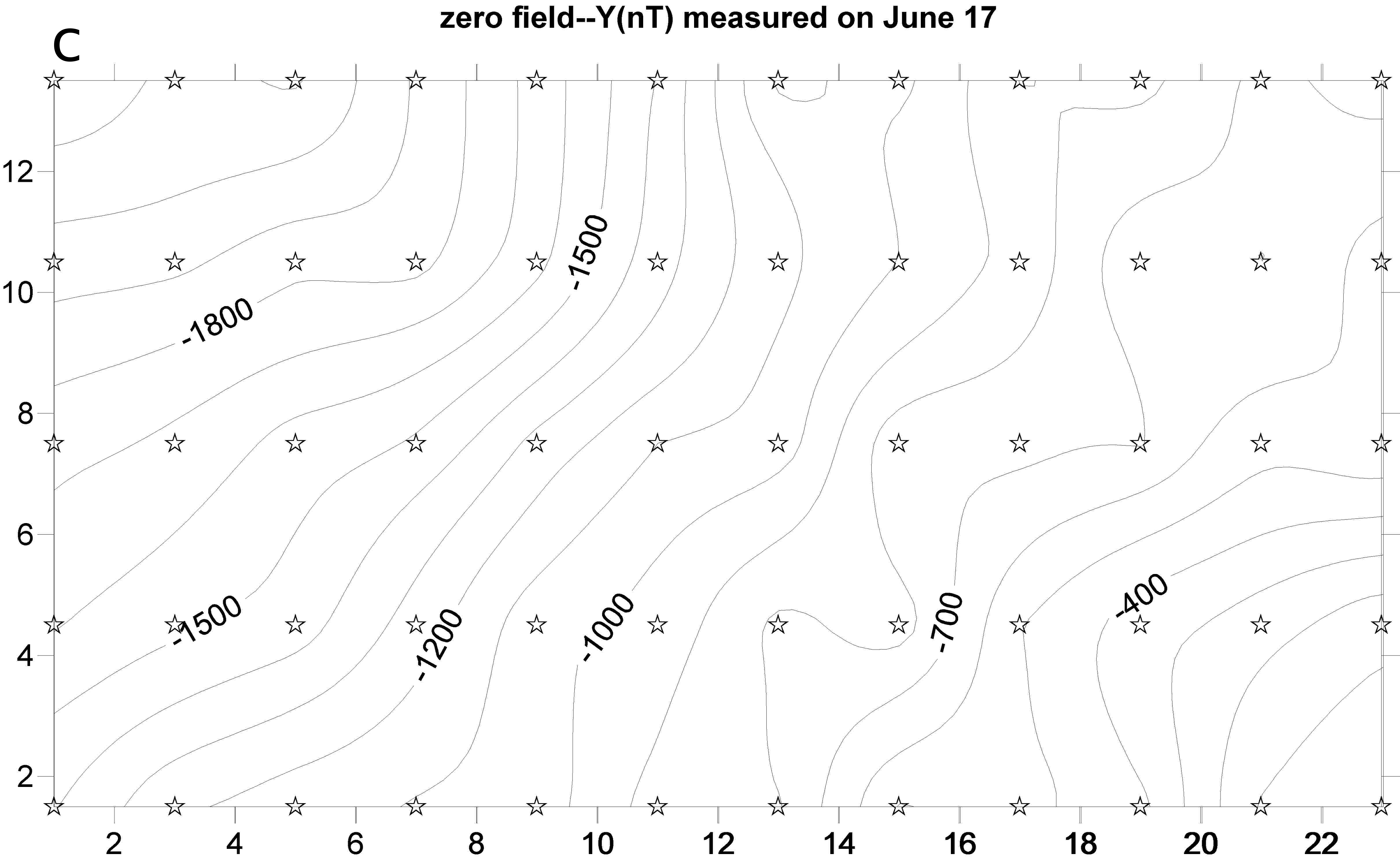

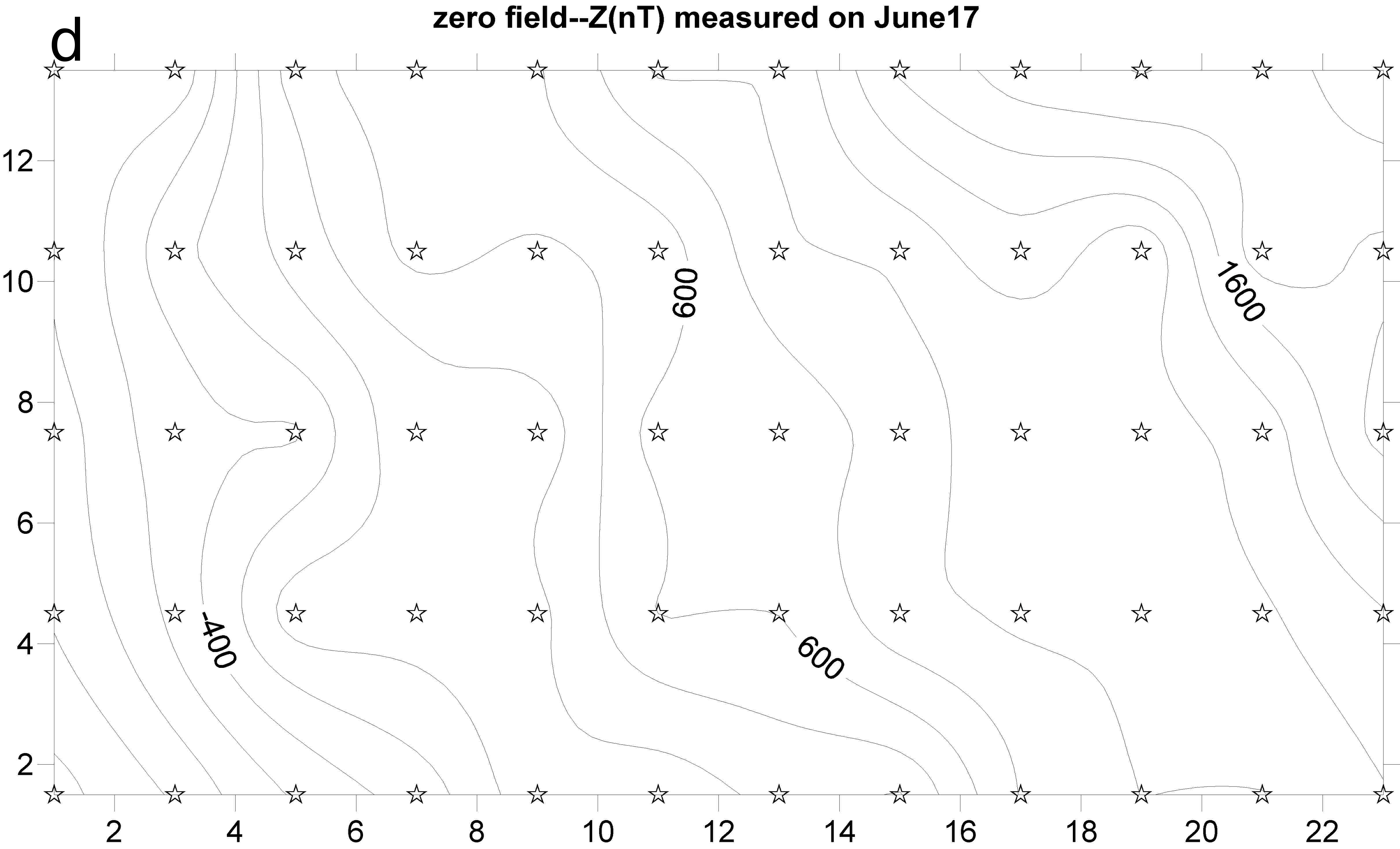


Figure 1 Zero field setup (a) and distribution of residual magnetic field with three components (b, c, d). The unit is nT. Numbers in horizontal and vertical axis were the dimension reference of the sediment area. Star symbols in the maps were the field measurement sites.

Table 1 An example to indicate large spatial variation in the sediment by comparing standard deviation and Poisson error of one averaged profile (average of 7 profiles).

| Depth (mm) | Mean | Standard deviation | Min. | Max. | Poisson error |
| --- | --- | --- | --- | --- | --- |
| 1 | 2.6 | 2.4 | 0 | 7 | 1.6 |
| 2 | 4.4 | 6.6 | 0 | 19 | 2.1 |
| 3 | 37.3 | 92.0 | 0 | 246 | 6.1 |
| 4 | 42.4 | 87.4 | 0 | 240 | 6.5 |
| 5 | 91.1 | 125.9 | 5 | 330 | 9.5 |
| 6 | 150.1 | 157.8 | 7 | 375 | 12.3 |
| 7 | 61.3 | 84.4 | 7 | 235 | 7.8 |
| 8 | 36.3 | 44.5 | 2 | 133 | 6.0 |
| 9 | 25.7 | 20.5 | 4 | 60 | 5.1 |
| 10 | 23.1 | 13.7 | 3 | 37 | 4.8 |
| 11 | 36.9 | 52.0 | 5 | 153 | 6.1 |
| 12 | 27.6 | 22.8 | 4 | 76 | 5.3 |
| 13 | 33.9 | 22.4 | 4 | 68 | 5.8 |
| 14 | 40.1 | 33.4 | 6 | 99 | 6.3 |
| 15 | 41.9 | 38.9 | 9 | 99 | 6.5 |
| 16 | 37.9 | 31.4 | 4 | 84 | 6.2 |
| 17 | 46.4 | 64.5 | 2 | 185 | 6.8 |
| 18 | 45.3 | 62.7 | 1 | 170 | 6.7 |
| 19 | 24.9 | 33.1 | 3 | 82 | 5.0 |
| 20 | 26.6 | 44.2 | 0 | 122 | 5.2 |
| 21 | 22.6 | 35.8 | 0 | 101 | 4.8 |
| 22 | 12.9 | 12.2 | 2 | 34 | 3.6 |
| 23 | 11.3 | 11.3 | 0 | 30 | 3.4 |
| 24 | 9.7 | 12.1 | 0 | 34 | 3.1 |
| 25 | 18.6 | 19.9 | 1 | 56 | 4.3 |

Table 2 A summary of MTB population density in Earth’s field, zero field and alternating field. Kolmogorov-Smirnov test accompanied with null hypothesis is used to test the difference of population density in zero field and alternating field from that in Earth’s field. If null hypothesis is rejected, the two datasets are seen different, otherwise are same. In principle population density in Earth’s field is larger than that in zero field and alternating field, which can be supported by rejection of null hypothesis (i.e. two datasets are different). Because after first and second zero-field settings, MTB took time to recover in Earth’s field, during which population density was still low, population density in Earth’s field was calculated based on the data italic format in Table S1 Data. Population density in zero field and alternating field was the average of all single profiles in corresponding field setting.

| **Field setting** | Cells/µl | Kolmogorov-Smirnov test | |
| --- | --- | --- | --- |
|  |  | 95% confidence level | Confidence level for rejecting null hypothesis |
| Bavaricum Earth field  Bavaricum zero field  Bavaricum alternating field | 14.6± 9.6  6.7 ± 4.3  7.5 ± 4.8 |   Rejected  Rejected |   99%  99% |
| Cocci Earth field  Cocci zero field  Cocci alternating field | 9.9 ± 6.8  10.5± 11.4  0.5± 0.8 |   Not Rejected  Rejected |   85%  99% |
